# Changes in the in vitro activity of platinum drugs when administered in two aliquots

**DOI:** 10.1186/s12885-016-2731-1

**Published:** 2016-08-26

**Authors:** Zaynab Al-Eisawi, Philip Beale, Charles Chan, Jun Qing Yu, Nicholas Proschogo, Mark Molloy, Fazlul Huq

**Affiliations:** 1 Discipline of Biomedical Science, Sydney Medical School, University of Sydney, Sydney, NSW 2141 Australia; 2 Sydney Cancer Centre, Concord Hospital, Sydney, NSW 2139 Australia; 3 Department of Pathology, Concord Hospital, Sydney, NSW 2139 Australia; 4 Mass Spectrometry Unit, School of Chemistry, University of Sydney, Sydney, NSW 2006 Australia; 5 Australian Proteome Analysis Facility, Macquarie University, Sydney, NSW 2109 Australia; 6 Department of Medical Laboratory Sciences, Faculty of Allied Health Science, Hashemite University, Zarqa, Hashemite Kingdom of Jordan; 7 Discipline of Biomedical Science, School of Medical Sciences, Sydney Medical School, The University of Sydney, Cumberland Campus C42, 75 East Street, Lidcombe, NSW 1825 Australia

**Keywords:** Cisplatin (CS), Carboplatin (CB), Oxaliplatin (OX), Drug combination, Synergism, Drug uptake, Drug resistance, Aging effect

## Abstract

**Background:**

The management of ovarian cancer remains a challenge. Because of the lack of early symptoms, it is often diagnosed at a late stage when it is likely to have metastasized beyond ovaries. Currently, platinum based chemotherapy is the primary treatment for the disease. However acquired drug resistance remains an on-going problem. As cisplatin brings about apoptosis by intrinsic and extrinsic pathways, this study aimed to determine changes in activity of platinum drugs when administered in two aliquots as against a bolus and sought to determine association with changes in GSH, speciation of platinum drugs and changes in protein expression.

**Methods:**

The efficacy of administering cisplatin, carboplatin and oxaliplatin in two aliquots with a time gap was investigated in ovarian A2780, A2780^cisR^, A2780^ZD0473R^ and SKOV-3 cell lines. The cellular accumulation of platinum, level of platinum − DNA binding and cellular glutathione level were determined, and proteomic studies were carried out to identify key proteins associated with platinum resistance in ovarian A2780^cisR^ cancer cell line.

**Results:**

Much greater cell kill was observed with solutions left standing at room temperature than with *freshly* prepared solutions, indicating that the increase in activity on *ageing* was related to speciation of the drug in solution. Proteomic studies identified 72 proteins that were differentially expressed in A2780 and A2780^cisR^ cell lines; 22 of them were restored back to normal levels as a result of synergistic treatments, indicating their relevance in enhanced drug action.

**Conclusions:**

The proteins identified are relevant to several different cellular functions including invasion and metastasis, cell cycle regulation and proliferation, metabolic and biosynthesis processes, stress-related proteins and molecular chaperones, mRNA processing, cellular organization/cytoskeleton, cellular communication and signal transduction. This highlights the multifactorial nature of platinum resistance in which many different proteins with diverse functions play key roles. This means multiple strategies can be harnessed to overcome platinum resistance in ovarian cancer. The results of the studies can be significant both from fundamental and clinical view points.

## Background

Platinum-based drugs cisplatin (CS), carboplatin (CB), and oxaliplatin (OX) are routinely used in the clinic to treat various cancers including testicular, ovarian, lung, bladder, colon, head and neck cancers [1]. However, efficacy is limited by dose limiting toxicities and acquired drug resistance [2] that may arise due to decreased cellular accumulation of platinum drugs, inactivation by conjugation with glutathione or sequestration involving metallothionein, enhanced tolerance to platinum-DNA adducts and enhanced DNA repair mechanisms [2, 3].

Cancer related pathways are bound to be highly complex often involving both intrinsic and extrinsic pathways [4]. As applied to cell death caused by CS, it was suggested that depending on the status of the cell, different pathways would become more significant at different time points. We proposed that the administration of first aliquot of CS would place cancer cells under increased oxidative stress caused by depletion of cellular thiols due to their binding with the drug [5] and if so when the second aliquot was administered after a brief time period (2 to 4 h), depleted glutathione level would allow more of the drug to bind with DNA resulting into increased apoptosis. Thus, the sequenced administration of CS in two aliquots with a small time gap could be looked upon as being the combination of two drugs with somewhat different mechanisms of action [5].

**Fig. 1:**
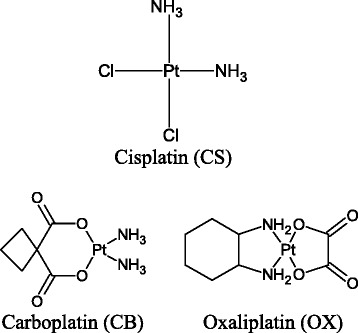
Chemical structures of cisplatin, carboplatin and oxaliplatin

The present study aimed to determine the efficacy of the administration of CS, CB and OX (Fig. 1) in two aliquots with time gaps of 2, 4, 8, 24 h in ovarian tumour models. We also sought to determine whether the use of ‘aged solutions’ of the drugs (where the solutions for the second aliquots were left standing at room temperature for the duration of the time gap) had a similar or greater effect on cell kill. The rationale behind using both *fresh* and *aged* solutions was to determine the effect of hydrolysis of platinum drugs on the combined drug action. Although platinum − DNA binding is believed to be an essential step in CS induced apoptosis, the programmed cell death is brought about downstream by multiple proteins. Thus, the study also aimed to determine changes in expression of key proteins associated with drug resistance in ovarian cancer cell lines.

## Methods

### Materials

CB and OX were obtained from Sigma Aldrich, Sydney, Australia. CS was synthesized according to previously described method [6]. Foetal calf serum (FCS), RPMI-1640, 200 mM L-glutamine, and 5.6 % sodium bicarbonate were obtained from Trace Biosciences Pty Ltd Australia. DNA extraction kit JETQUICK Blood DNA Spin Kit/50 was obtained from Astral Scientific Pty Ltd, Sydney, Australia. GSH/GSSG-Glo™ assay kit was obtained from Promega, Sydney, Australia. Other chemicals were obtained mostly from Sigma-Aldrich, Sydney, Australia. Ovarian cancer A2780, A2780^cisR^, A2780^ZD0473R^ and SKOV-3 cell lines were gifts from Ms. Mei Zhang, Royal Prince Alfred Hospital, Sydney, Australia. Stock solutions of platinum drugs were prepared to a final concentration of 1 mM; CS was first dissolved in DMF then made up in milli-Q water to a final ratio of 1:4 DMF to milli-Q water, whereas CB and OX were prepared in milli-Q water only. Stock solutions were then filtered to insure sterility.

### Cell culture

Human ovarian cancer A2780, A2780^cisR^, A2780^ZD0473R^ and SKOV-3 cell lines (Table 1) were seeded in 25 cm^2^ tissue culture flasks in an incubator at 37 °C in a humidified atmosphere consisting of 5 % CO_2_ and 95 % air. The cells were maintained in logarithmic growth phase in complete medium consisting of RPMI 1640, 10 % heat inactivated FCS, 20 mM Hepes, 0.11 % bicarbonate, and 2 mM glutamine without antibiotics [7]. Each cell line was seeded at a density of 4–6 × 10^3^ cells/well in flat-bottomed 96-well culture plate in 10 % FCS/RPMI 1640 culture medium. The plate was incubated for 24 h at 37 °C in a humidified atmosphere allowing cells to attach.

**Table 1:** Human ovarian cancer cell lines used in this study

Cell line	Phenotype
A2780	Untreated ovarian tumour
A2780^cisR^	CS resistant ovarian tumour
A2780^ZD0473R^	ZD0473^a^ resistant ovarian tumour
SKOV-3	Oestrogen receptor positive ovarian tumour

^a^ZD0473 (also known as JM473 and AMD 473) is a sterically hindered platinum complex with a *cis*-geometry like cisplatin

### Cytotoxicity assay

MTT reduction assay was carried out to determine cytotoxicity of CS, CB and OX administered as a bolus and in two aliquots with a time gap. Stock solutions of drugs were subjected to serial dilutions to give final concentrations ranging from 0.16 to 250 μM. The dilutions were performed using 10 % RMPI-1640 medium without serum as the vehicle and were added to equal volumes of cell culture in triplicate wells and then cells were left to incubate for 72 h. These treatments were carried out to determine IC_50_ values i.e. drug concentrations required for 50 % cell kill. In treatment in two aliquots with a time a gap, cells were treated with solutions of CS, CB and OX at three different concentrations based on their IC_50_ values. The first aliquot administered at time zero was that of a freshly prepared solution (denoted as ‘*fresh’*) whereas the second aliquot administered at 2 h (0/2 h), 4 h (0/4 h), 8 h (0/8 h) or 24 h (0/24 h) was using either freshly prepared solution (*fresh*) so that the combination was termed *fresh/fresh* or aged solution left at room temperature for the period of the time gap (denoted as ‘*aged*’) so that the combination was termed *fresh/aged*. Cells in drug free medium served as control. The rationale behind doing experiments with both *fresh* and *aged* solutions was to determine the effect of hydrolysis of platinum drugs on the combined drug action. The period of drug treatment was 72 h counted from the time of administration of the first dose. Cell growth inhibition was determined using the MTT reduction assay. Combination index values (CIs) were used as measures of synergism, additiveness or antagonism calculated using the program CalcuSyn [8–10] and previously described method [11].

### Platinum accumulation and platinum-DNA binding

Cellular accumulation of platinum and platinum − DNA binding levels in A2780 and A2780^cisR^ cell lines were determined as applied to administration of CS in two aliquots with a time gap of 2 and 4 h and at a final concentration of 50 mM, for both *fresh/fresh* and *fresh*/*aged* combinations. The drug was added to culture plates containing exponentially growing A2780 and A2780^cisR^ cells in 10 ml 10 % FCS/RPMI 1640 culture medium (cell density = 5 × 10^6^ cells/ml). The cells containing the drug were incubated for 24 h at the end of which cell monolayers were collected and cell suspensions (10 ml) were transferred to centrifuge tube and spun at 3500 rpm for 2 min at 4 °C. The cells were washed twice with ice-cold phosphate-buffered saline (PBS) and the pellets were stored at −20 °C until assayed. At least three independent experiments were performed.

### Cellular accumulation

Following drug incubation the cell pellets were suspended in 0.5 ml 1 % triton-X, held on ice then sonicated. Total intracellular platinum contents were determined by graphite furnace atomic absorption spectrophotometry.

### Drug–DNA binding

High molecular weight DNA from cell pellets were isolated using JETQUICK Blood DNA Spin Kit/50 (Astral Scientific, Australia) according to the modified protocol of Bowtell [12]. Platinum contents of the samples were determined by graphite furnace AAS. A_260_/A_280_ ratios were found to be between 1.75 and 1.8 for all samples, indicating high purity of the DNA.

### Cellular glutathione

As a measure of cellular health and the redox state of the cells, the levels of total glutathione (GSH and GSSG) and oxidised glutathione (GSSG) in A2780 and A2780^cisR^ cell lines were determined as applied to treatments with CS and CB administered in two aliquots with a time gap of 4 h. Drugs made in 10 % RMPI-1640 serum free medium were added to equal volumes of cell culture wells of a white wall clear bottom 94 well plate containing exponentially growing A2780 and A2780^cisR^ cells (cell density = 12 × 10^3^ cells/well). Cells were left to incubate for 24 h. The medium was aspirated out of the treatment wells with minimal disturbance of the cell pellets and cells were washed with 200 μl of PBS, then the levels of glutathione were determined using the GSH*/*GSSG*-*Glo™ Assay kit (Promega, Australia). The plate was read in a LUMIstar Omega luminometer (BMG LABTECH, USA).

### Mass spectral analysis

Mass spectrometry was used to explore hydrolysis of OX rather than that of CS as OX and its hydrolysis products were more sensitive to mass spectral measurements than those of CS. Solution of OX first made in milli-Q water, was diluted (1:2) with methanol, cell culture medium, or pH adjusted (neutral) milli-Q water. The solutions were injected by syringe pump (flow rate 150 ul/h) into a Bruker Apex Qe 7 T Fourier Transform Ion Cyclotron Resonance Mass Spectrometer (FTICR) in positive ion electrospray ionization mode. The instrument was optimized and externally mass calibrated before use. The presence and evolution of OX species was monitored over a period of 4 h, with measurements made every hour.

### Proteomics

Proteomic studies were carried to determine the proteins that were differentially expressed in the parent A2780 and cisplatin-resistant A2780^cisR^ cell lines but were restored back due to treatment with CS in two aliquots. Ovarian cancer A2780 and A2780^cisR^ cell lines were cultured in 50 cm^2^ petri dishes to produce at least a million cells per dish. Cells were treated with solutions of CS (at IC_50_) administered as a bolus and in two aliquots with 2 h time gap using both *aged* and *fresh* solutions (CS + CS (2/0 h) *aged/aged* and CS + CS (2/0 h) *fresh/fresh*). Untreated control cells were also included. The period of incubation with the drugs was 24 h. Following drug treatment, cell pellets were collected, rinsed with ice-cold PBS and centrifuged at 3500 rpm for 2 min at 4 °C. The pellets were lysed in a cell lysis solution containing 2 M thiourea, 8 M urea, 4 % CHAPS, 65 mM dithiothreitol (BIORAD, Australia). Isoelectric focusing (IEF) of the sample containing 200 μg of proteins was performed using 11 cm, pH 3–10 non-linear ReadyStrip™ IPG Strip in Protean i12 IEF cell unit (BIORAD, Australia) rehydrated in 8 M urea, 2 M thiourea, 4 % CHAPS, 60 mM dithiothreitol, 0.2 % carrier ampholyte, 0.0002 % bromophenol blue and deStreak (BIORAD, Australia). Two equilibration steps of the IPG were performed in SDS equilibration buffer containing SDS, 6 M urea, 50 % glycerol, 1.5 M Tris HCl (pH 8.8), and bromophenol blue with the first containing 0.5 g dithiothreitol and the second 0.5 g iodoacetamide. Protein concentration was determined using Bio-Rad Protein Assay (BIO-RAD, Australia). SDS-PAGE was performed using 4–20 % SDS Criterion™ TGX™ pre-cast gels in a Criterion Dodeca™ cell separation unit (BIO-RAD, Australia) at constant 200 V for 100 min in a Tris-glycine-HCl buffer system. The gels were stained with Bio-Safe Coomassie Stain (BIO-RAD, Australia) for 60 min. At least a duplicate of gels containing protein spots from the same sample were used for analysis. The gel images obtained by ChemiDoc™ MP Imaging system (BIO-RAD, Australia) were analysed for protein spots using Melanie version 7.0 software (GeneBio, Switzeland). A 2.0 fold change in the expression of a protein across the matched groups was used as the cut-off for differential expression. Analysis of variance (ANOVA), a statistical tool used to detect differences between experimental group means, was performed using a target significance level of 0.05.

### MALDI-TOF/TOF MS

Protein spots were excised from preparative 2-D gels stained with Bio-Safe Coomassie Stain (BIO-RAD, Australia). The spots were destained with 120 μl of (50 % acetonitrile (ACN)/50 mM NH_4_HCO_3_) solution and heated at 37 °C for 30 min with mild shaking. The solution was then discarded. The gels were treated with 25 μl ACN and left to dry for 15 min. The solution was discarded then the spots were left to dry with the lid left open in the oven at 37 °C for 15 min followed by cooling at 4 °C. The spots were digested with 10 μl trypsin for 10 min on ice. The trypsin supernatants were placed in 96-well plate at 4 °C followed by 10 μl addition of 25 mM NH_4_HCO_3_ for overnight digestion at 37 °C. The resulting peptides were extracted with 0.1 % trifluoroacetic acid (TFA) then extracted and concentrated by C18 zip-tips (Millipore, μ-C18, P10 size) on Xcise (Proteome Systems). A 1 μl aliquot was manually spotted onto a MALDI AnchorChip plate with 1 μl of matrix (CHCA, 1 mg/mL in 90 % v/v ACN, 0.1 % TFA) and left to dry in air. Matrix assisted laser desorption ionisation mass spectrometry (MALDI-MS) was performed with 4800 plus MALDI TOF/TOF Analyser (AB Sciex). A neodymium-doped yttrium aluminum garnet (Nd:YAG) laser (355 nm) was used to irradiate the sample. Spectra were acquired in reflectron MS scan mode in the mass range of 700–4000 Da. The instrument was then switched to MS/MS (TOF-TOF) mode where the eight strongest peptides from the MS scan were isolated and fragmented by collision induced dissociation (CID), then re-accelerated to measure their masses and intensities. A near point calibration was applied and would give a typical mass accuracy of 50 ppm or less. The data on peptides masses were analysed using database search program Mascot (Matrix Science Ltd, London, UK). The peak lists were searched against *Homo sapiens* entries in the SwissProt database. The protein identification was undertaken at Australian Proteome Analysis Facility (APAF) the infrastructure provided by the Australian Government through the National Collaborative Research Infrastructure Strategy (NCRIS).

## Results

### Cytotoxicity

**Fig. 2:**
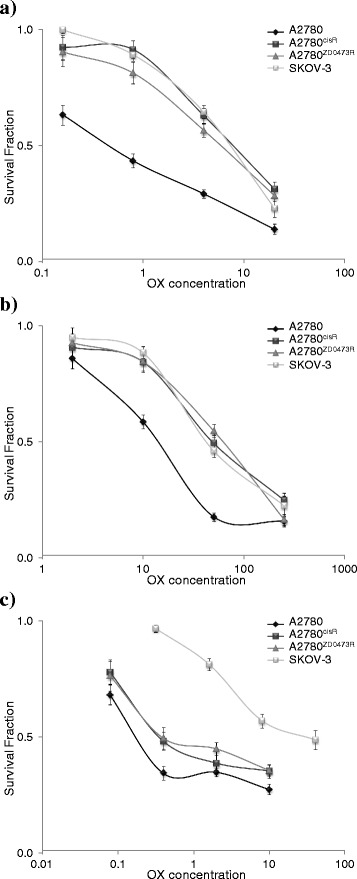
Cell growth inhibition following increasing concentrations of platinum drugs. Cell survival fractions of ovarian cancer A2780, A2780^cisR^, A2780^ZD0473R^ and SKOV-3 cell lines following treatment with increasing concentrations of **a** CS, **b** CB and **c** OX for 72 h were determined using MTT assay and spectrophotometric measurement. Error bars represent the standard deviation (where straight lines or curves containing the dot points are meaningless)

Figure 2 shows the cell survival fractions versus concentration plots for CS, CB, and OX as applied to the human ovarian cancer A2780, A2780^cisR^, A2780^ZD0473R^ and SKOV-3 cell lines. The IC_50_ values of CS, CB and OX are presented in Table 2. As expected, the values were higher in the resistant A2780^cisR^, A2780^ZD0473R^ and SKOV-3 cell lines with OX having the largest value in SKOV-3. The parent A2780 cell line was most sensitive to the drugs, A2780^ZD0473R^ was least sensitive to CB and SKOV-3 was least sensitive to OX.

**Table 2:** Summary of the IC_50_ values (μM) and resistance factors (RF) for CS, CB and OX as applied to the ovarian cancer A2780, A2780^cisR^, A2780^ZD0473R^ and SKOV-3 cell lines. IC_50_ is the drug concentration required for 50 % cell kill and RF is the ratio of the IC_50_ value in the resistant A2780^cisR^ and A2780^ZD0473R^ cell lines over that in the responsive parent A2780 cell line

	A2780	A2780^cisR^	RF	A2780^ZD0473R^	RF	SKOV-3
CS	0.5 ± 0.03	8.2 ± 0.6	17.7	6.0 ± 0.5	13.0	10.2 ± 0.5
CB	14.0 ± 1.4	48.9 ± 3.9	3.5	64.6 ± 3.2	4.6	43.4 ± 3.9
OX	0.2 ± 0.01	0.4 ± 0.02	1.9	0.4 ± 0.03	2.1	43.6 ± 3.0

### Administration in two aliquots

Figure 1 gave the combination index (CI) values applying to administration of CS, CB and OX in two aliquots with a time gap of 2, 4, 8, or 24 h to the ovarian cancer A2780, A2780^cisR^, A2780^ZD0473R^ and SKOV-3 cell lines where CI values of <1, =1 and >1 indicated respectively synergism, additivity and antagonism in combined action. Cells were treated with solutions of CS, CB and OX at three different concentrations based on their IC_50_ values. When CS was administered in two aliquots (*fresh*/*aged*), the killing of A2780 cells was most pronounced when the time gap was 8 h; all other time gaps were found to be additive to antagonistic. In A2780^cisR^ cell line, much pronounced cell kill was observed for time gaps of 2, 4 and 8 h and antagonism was observed when it was increased to 24 h. In A2780^ZD0473R^ cell line also, extremely pronounced cell kill was observed for time gaps of 2, 4 and 8 h and reduced cell kill was observed when this was increased to 24 h. In SKOV-3 cell line, synergistic cell kill was observed only for the 24 h time gap. When CB was administered in two aliquots (*fresh*/*aged*) in A2780 cell line synergistic kill was observed when the time gap was 4, 8 or 24 h. It was most pronounced when the time gap was 4 and 8 h but antagonistic when the time gap was 2 h. In A2780^cisR^ cell line, pronounced cell kill was observed for the time gaps of 2, 4 and 8 h and reduced cell kill was observed when it was 24 h. In A2780^ZD0473R^ cell line also, extremely pronounced cell kill was observed for time gaps of 2, 4 and 8 h and reduced cell kill was observed when this was increased to 24 h. In SKOV-3 cell line, antagonism was observed for all time gaps.

**Fig. 3:**
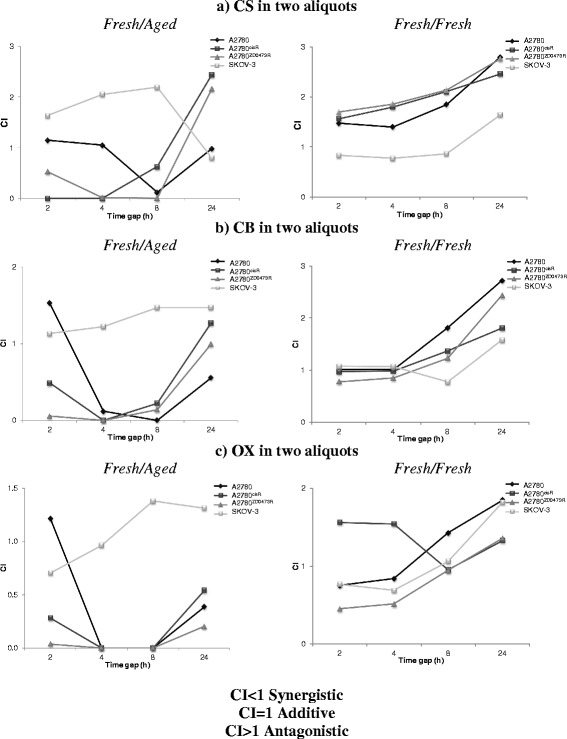
Combination Index (CI) values following the administration of **a** CS, **b** CB and **c** OX in two aliquots with a time gap of 2, 4, 8, or 24 h as applied to the ovarian cancer A2780, A2780^cisR^, A2780^ZD0473R^ and SKOV-3 cell lines using both *fresh/fresh* and *fresh/aged* combinations. CI values were calculated following 72 h treatments. CI values of <1, =1 and >1 indicate respectively synergism, additivity and antagonism in combined drug action

When OX was administered in two aliquots (*fresh*/*aged), c*ell kill in A2780 cells was pronounced when the time gap was 4, 8 and 24 h and reduced cell kill was observed when the time gap was 2 h. In A2780^cisR^ and A2780^ZD0473R^, all the time gaps (especially 4 and 8 h) produced pronounced cell kill. When OX was administered in two aliquots (*fresh/aged*) to SKOV-3 cell line, cell kill was pronounced when the time gap was 2 h, additive when it was 4 h and reduced cell kill was observed when it was increased to 8 and 24 h. The results indicate the administration of CS, CB, and OX in two aliquots, with the first aliquot *fresh* and the second aliquot *aged,* generally caused enhanced cell kill especially in the resistant A2780^cisR^ and A2780^ZD0473R^ cell lines. In contrast, administration of CS, CB and OX in two aliquots with both being *fresh*, produced mostly reduced cell kill in A2780, A2780^cisR^ and A2780^ZD0473R^ cell lines. In SKOV-3 cell line, enhanced cell kill resulted when the time gap was 2, 4, and 8 h and reduced cell kill resulted when it was 24 h (Fig. 3).

### Platinum accumulation

**Fig. 4:**
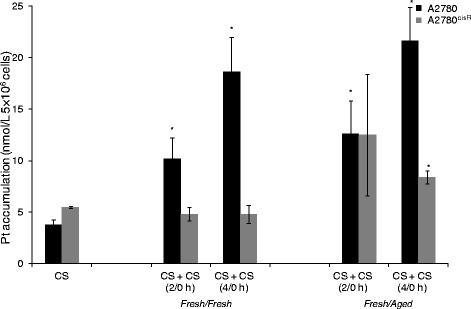
Cellular platinum accumulation in ovarian cancer A2780 and A2780^cisR^ cell lines resulting from administration of CS in two aliquots using both *fresh/fresh* and *fresh/aged* combinations of CS with a time gap of 2 and 4 h. Cells were treated with the drugs for 24 h followed by collection, lysis and finally Pt was determined using AAS. Data was statistically analyzed using the paired Student’s *t* test: * *p* < 0.05 indicates significant difference from control. Error bars represent the standard deviation

To determine whether the enhanced cell kill was associated with an increase in platinum accumulation, the levels of platinum accumulation following the administration of CS in two aliquots with a time gap of 2 and 4 h in A2780 and A2780^cisR^ cell lines were determined, using both *fresh* and *aged* solutions (Fig. 4). It was found that both *fresh/fresh* and *fresh*/*aged* combinations were associated with significantly higher platinum accumulations in A2780 cell line. The highest platinum accumulation resulted from *fresh*/*aged* combination with 4 h time gap. In the resistant A2780^cisR^ cell line, only the *aged* solution resulted in increased platinum accumulation.

### Platinum-DNA binding

As a key step in the antitumour action of platinum drugs is their binding with DNA (that initiates downstream processes in the cell cycle leading to apoptosis), platinum − DNA binding levels in A2780 and A2780^cisR^ cell lines were determined applying to administration of solutions of CS in two aliquots with a time gap of 2 and 4 h, using both *fresh/fresh* and *fres*h/*aged* combinations. Figure 2 gave platinum − DNA binding levels in ovarian cancer A2780 and A2780^cisR^ cell lines as applied to the administration of CS in two aliquots with a time gap of 2 and 4 h.

**Fig. 5:**
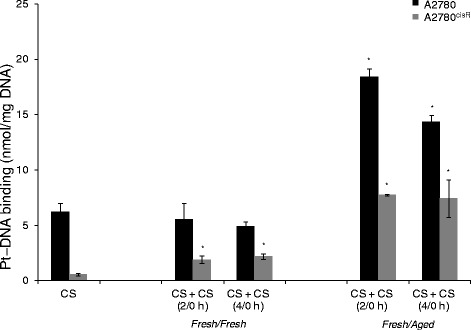
Platinum − DNA binding in ovarian cancer A2780 and A2780^cisR^ cell lines as applied to the administration of CS in two aliquots using both *fresh/fresh* and *fresh/aged* combinations of CS with a time gap of 2 and 4 h. Cells were treated with the drugs for 24 h followed by collection, DNA extraction and finally pt detection using AAS. Data was statistically analyzed using the paired Student’s *t* test: * *p* < 0.05 indicates significant difference from control. Error bars represent the standard deviation

The platinum − DNA binding levels in A2780^cisR^ cell line were generally found to be greater from administration in two aliquots than from the bolus for both 2 and 4 h time gaps, as applied to both *fresh/fresh* and *fresh/aged* combinations but more so for the latter. In the parent A2780 cell line, only the *fresh/aged* combination resulted in greater Pt − DNA binding level (for both 2 and 4 time gaps) than the bolus (Fig. 5).

### Cellular glutathione

**Fig. 6:**
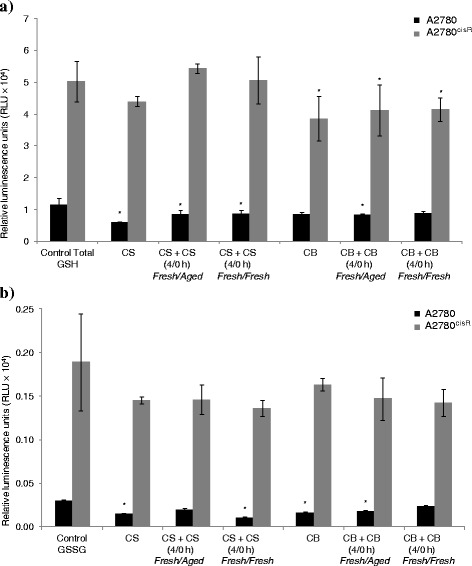
Levels of **a** total glutathione (GSH plus GSSG) and **b** oxidized glutathione (GSSG) in relative luminescence units (RLU × 10^4^) in A2780 and A2780^cisR^ cells before and after their treatments with solutions of CS and CB administered as a bolus and in two aliquots with time gaps of 2 and 4 h using *fresh/fresh* and *fresh/aged* combinations. Cells were treated for 24 h and glutathione content was determined using GSH/GSSG-Glo Assay kit. Data was statistically analyzed using the paired Student’s *t* test: * *p* < 0.05 indicates significant difference from control. Error bars represent the standard deviation

Since oxidative stress is like a double edged sword in cancer that can lead to both programmed cell death and cell survival, the effect of nature of administration of CS and CB on the cellular glutathione level was also investigated. Specifically, the effect of administration of the drug as a bolus and in two aliquots applying to both *fresh/fresh and fresh/aged* solutions of CB and CS was investigated. The levels of total glutathione (GSH plus GSSG) and the oxidized glutathione (GSSG) in A2780 and A2780^cisR^ cell lines were determined (Fig. 6a, b). In line with reported result [13–15], total glutathione was found to be higher in the CS-resistant A2780^cisR^ cell line than in the parent A2780 cell line before and after drug treatment. There was a significant decrease in total glutathione level following treatment of A2780 and A2780^cisR^ cells with solutions of CS and CB. This was found to be true for both bolus administration and that in two aliquots but more so for the bolus. The results indicate that bolus administration of CS and CB produced a greater oxidative stress in the cells than that in two aliquots. As noted earlier, there was an increase in cell kill due to the administration of CS and CB in two aliquots using *fresh/aged* combinations.

### Platinum speciation

**Fig. 7:**
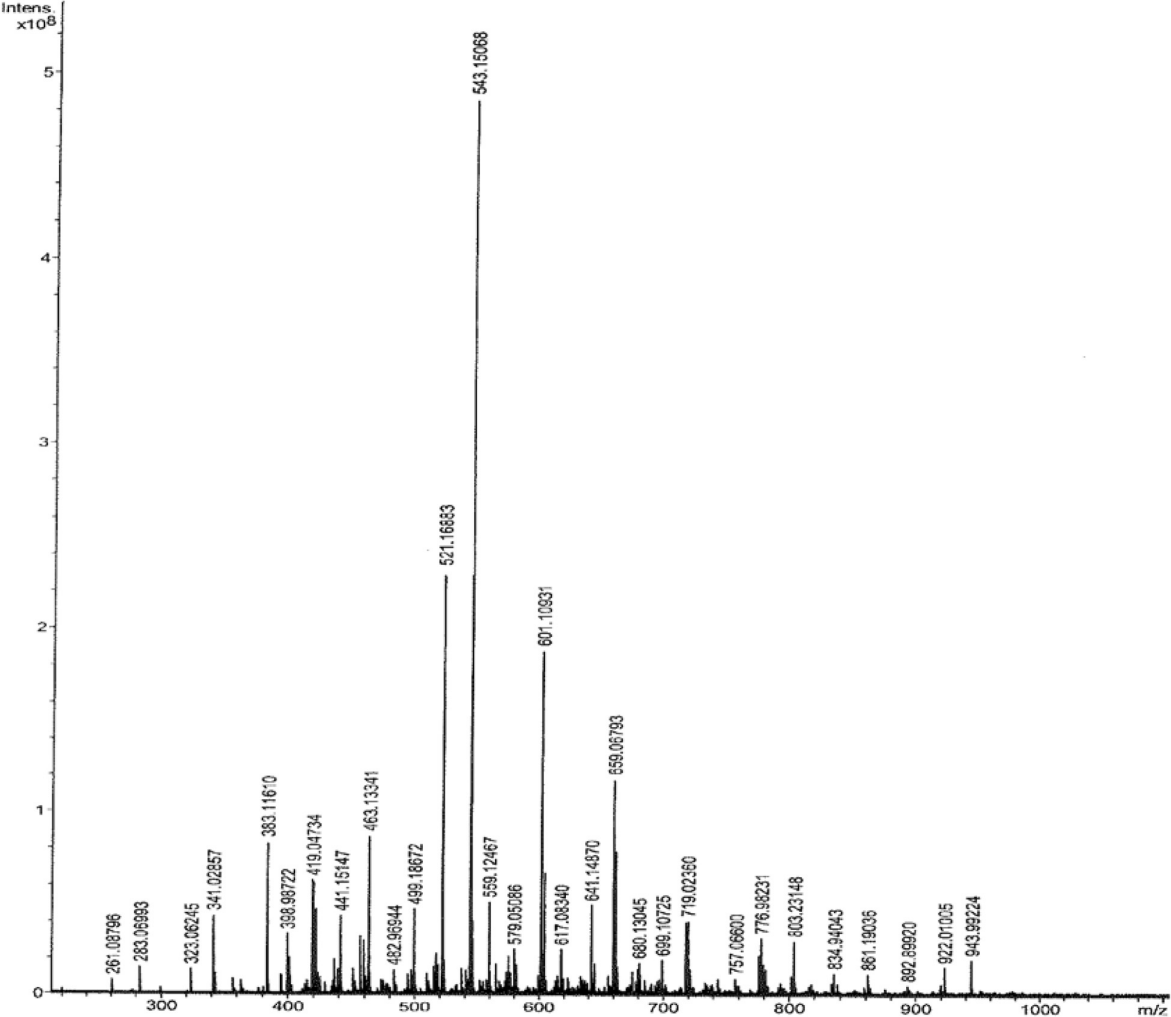
Magnified mass spectrum showing major peaks of OX dissolved in cell culture medium. The 543.1 peak is the base peak due to the cell culture medium

With the idea that the greater cell kill resulting from administration of CS, CB and OX in two aliquots using *fresh/aged* combinations could be due to speciation of the drugs (resulting in the production of more cytotoxic species), mass spectral analysis of solutions of OX in cell culture media were performed. Figure 7 gave magnified mass spectrum showing major peaks of OX dissolved in cell culture medium. The major peaks observed in the mass spectra are given in Table 3.

**Table 3:** Major peaks in the mass spectra of *aged* solution of OX in cell media

m/z	No. of possibilities	Formula	Relative intensity (×10^6^)	Suggested structure
398	1	Pt(C_6_H_14_N_2_)(C_2_O_4_)	16	
420.0493	4	[Pt(C_6_H_14_N_2_)(C_2_O_4_) + Na]^+^	4.6	
436.0233	2	Pt(C_6_H_14_N_2_)_2_ – 3H	7.2	
478.0080	14	[Pt(C_6_H_14_N_2_)(C_8_H_11_NO_3_)]^+^	8	
680.1303	67	[Pt(C_6_H_14_N_2_)(C_10_H_17_N_3_O_6_S)(CH_3_CH_2_SH) + 2H]^+^	26	
738.0889	157	[Pt(C_6_H_14_N_2_)(C_10_H_17_N_3_O_6_S)(C_3_H_7_NO_2_S)]^+^	9	
795.0480	277	[Pt_2_(C_6_H_14_N_2_)_2_(C_2_O_4_)_2_ + H]^+^	21	
940.2129	377	[Pt(C_6_H_14_N_2_)(C_10_H_17_N_3_O_6_S)_2_O]^+^	19	

Peak with m/z = 420.0493 is believed to be due to the molecular ion [Pt(DACH)(C_2_O_4_) + Na]^+^ where Na^+^ is from the culture media. Peak with m/z = 436.0233 is believed to be due to the molecular ion [Pt(DACH)(C_2_O_4_) + K]^+^ where K^+^ is from the culture media. Peak with m/z = 478.0080 is due to Pt(DACH)(Pyr) (where Pyr = pyridoxine (vitamin B_6_)), believed to be formed in the cell culture media. The peak with m/z = 680.1303 may be due to Pt(DACH)(SCH_3_)(GSH) formed in the culture media and that with m/z = 795.0480 is believed to be due to dimeric species consisting of two Pt(DACH) units catenated by two oxalate ligands. Finally, the peak with m/z = 940.2129 is believed to be due to Pt(DACH)(GSH)_2_.

### Proteomics

**Fig. 8:**
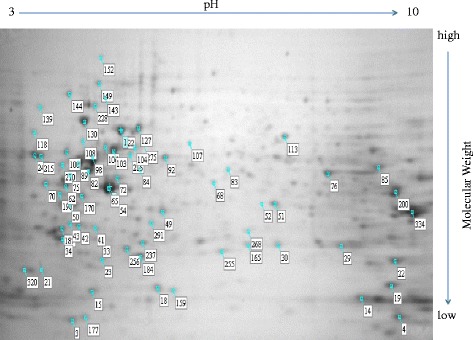
2-DE pattern of whole-cell proteins in A2780 cell line. The 2-D gel was stained with coomasiee brilliant blue (11 cm, pH 3–10 non-linear, 4–20 % SDS-PAGE, 200 μg proteins). The protein spots differentially expressed in A2780^cisR^ compared to the parent A2780 cell line identified in this study are marked with their spot number ID

**Fig. 9:**
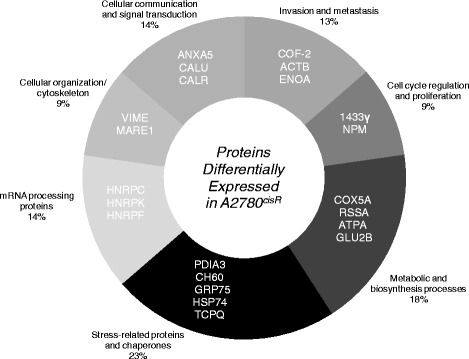
Grouping of proteins and enzymes based on the cellular functions that were found to be differentially expressed in the resistant A2780^cisR^ cell line as compared to the parent A2780 cell line and that have undergone further changes in expression after treatment with the administration of CS in two aliquots

As stated earlier, proteomic studies were carried out to identify the proteins differentially expressed in the resistant A2780^cisR^ cell line as compared to the levels found in parent A2780 cell line. 2-D gels resolved over 390 proteins of which 72 were found to be differentially expressed in A2780^cisR^ cell line as compared to the parent A2780 cell line (Fig. 8). Administration of CS in two aliquots with a time gap was found to restore the expression of at least 22 proteins to the levels found in the parent cell line, of which 12 were down-regulated and 10 up-regulated prior to drug treatment. A summary of the proteins, their possible functions, and associations with neoplasia are given in Table 4. The proteins belong to seven groups based on cellular functions namely: invasion and metastasis, cell cycle regulation and proliferation, metabolic and biosynthesis processes, stress-related proteins and chaperones, mRNA processing proteins, cellular organization/cytoskeleton, cellular communication and signal transduction (Fig. 9). The proteins are believed to be associated with platinum resistance in ovarian cancer. A more detailed description of their functions as applied to platinum resistance in ovarian cancer is given in the discussion.

**Table 4:** In total, 22 proteins with differential expression (2-fold increase or decrease, ANOVA *p* < 0.05) between A2780^cisR^ and the parent A2780 cell line due to treatment with CS in two aliquots with a time gap of 2 h using *fresh/fresh* and *fresh/aged* combinations were identified by MALDI-TOF/TOF

Spot	ID	Full name	MALDI	location	Expression in A2780^cisR^	Post treatment expression			Function	Tumour association
						CS	CS + CS			
							*fresh*	*aged*		
3	COX5A	Cytochrome c oxidase subunit 5A, mitochondrial	Score: 63 Mass: 16752 *pI*: 6.30 Coverage: 11 %	Mitochondria	Down Regulated	PR	PR	OR	Ribosome biogenesis; mitochondrial respiratory chain	Breast [77]; cervix [78]; colon [77]; gastric [31]; Kidney [79]; Lung [80–82]; Nasopharyngeal [32]; Oesophageal [77]; Ovarian [77]; Prostate [77]; Thyroid [83]
19	COF2	Cofilin‐2	Score: 127 Mass: 18725 *pI*: 7.66 Coverage: 39 %	Cytoplasm, cytoskeleton	Down Regulated	R	R	OR	Actin polymerization	Pancreatic [84]; Prostate [85]
34	1433G	14‐3‐3 protein gamma	Score: 315 Mass: 28285 *pI*: 4.80 Coverage: 44 %	Cytoplasm	Down Regulated	PR	PR	OR	Adapter protein	Breast [86]
41	MARE1	Microtubule‐associated protein RP/EB family member 1	Score: 62 Mass: 29980 *pI*: 5.02 Coverage: 29 %	Cytoplasm	Down Regulated	-	R	OR	Microtubule cytoskeleton dynamics; cell migration.	Colon [87]; lung [88]; gastric [89]; oesophageal [90];
50	NPM	Nucleophosmin	Score: 68 Mass: 32555 *pI*: 4.64 Coverage: 26 %	Nucleoplasm	Down Regulated	-	R	OR	Chaperone; ribosome biogenesis; p53 and ARF regulation	Bladder [91]; breast [92]; Colon [93]; gastric [94]; haematopoietic [40]; ovarian [95]; prostate [96]
51	ANXA1	Annexin A1	Score: 369 Mass: 38690 *pI*: 6.57 Coverage: 53 %	Nucleus, cytoplasm	Down Regulated	R	R	OR	Calcium/phospholipid-binding protein	breast [97]; gastric [98]; HNSC [36]; lung [99]; oesophageal [100, 101]; prostate [100, 102];
62	RSSA	40S ribosomal protein SA	Score: 311 Mass: 32833 *pI*: 4.79 Coverage: 34 %	Cell membrane, cytoplasm, nucleus	Up Regulated	R	R	-	laminin receptor; fate determination; tissue morphogenesis	Breast [103]; cervical [104]; colon [54, 55, 105]; melanoma [106]
65	ACTB	Actin, cytoplasmic 1	Score: 767 Mass: 41710 *pI*: 5.29 Coverage: 53 %	Cytoplasm	Up Regulated	PR	R	PR	cell motility	Colon [107]; gastric [108]; liver [109]; sarcoma [110]
70	CALU	Calumenin	Score: 141 Mass: 37084 *pI*: 4.47 Coverage: 14 %	Sarcoplasmic, endoplasmic reticulum	Up Regulated	-	OR	PR	Vitamin K-dependent carboxylation	Colon [111]; gastric [112]; glioblastoma [113]; prostate [114];
72	HNRPF	Heterogeneous nuclear ribonucleoprotein F	Score: 342 Mass: 45643 *pI*: 5.38 Coverage: 33 %	Nucleus	Down Regulated	R	OR	R	processing of pre-mRNAs; alternative splicing events	Gastric [115]; thyroid [116]
76	ENOA	Alpha‐enolase	Score: 471 Mass: 47139 *pI*: 7.01 Coverage: 47 %	Cytoplasm	Down Regulated	-	PR	R	Glycolysis; hypoxia tolerance; tumour suppressor	Breast [117]; lung [118]; nasopharyngeal [119];
85	ATPA	ATP synthase subunit alpha, mitochondrial	Score: 391 Mass: 59714 *pI*: 9.16 Coverage: 29 %	Mitochondria	Up Regulated	-	OR	OR	Production of ATP from ADP	Breast [120]; colon [121]; gastric [122]; leukemia [123]; thyroid [124];
92	PDIA3	Protein disulfide-isomerase A3	Score: 525 Mass: 56747 *pI*: 5.98 Coverage: 54 %	Endoplasmic reticulum	Down Regulated	PR	PR	R	endopeptidase; electron carrier;	Lung [125]; ovarian [48]; Prostate [46]; retinoblastoma [126]
100	HNRPK	Heterogeneous nuclear ribonucleoprotein K	Score: 123 Mass: 50944 *pI*: 5.39 Coverage: 26 %	Cytoplasm, nucleus	Up Regulated	-	OR	-	Pre-mRNA-binding proteins; p53 response to DNA damage	Breast [127]; colon [128]; Leukemia [129, 130]; lung [131]; Oropharyngeal [132]; pancreatic [133];
103	CH60	60 kDa heat shock protein, mitochondrial	Score: 762 Mass: 61016 *pI*: 5.70 Coverage: 39 %	Mitochondria	Up Regulated	PR	OR	PR	macromolecular assembly; stress working chaperone	Bronchus [134]; Colon [135]; leukemia [136]; prostate [137]
104	TCPQ	T‐complex protein 1 subunit theta	Score: 200 Mass: 59583 *pI*: 5.42 Coverage: 24 %	Cytoplasm	Down Regulated	R	OR	R	chaperone	Colon [138]; liver [139];
127	GRP75	Stress‐70 protein, mitochondrial	Score: 839 Mass: 73635 *pI*: 5.87 Coverage: 33 %	Mitochondria, nucleus	Down Regulated	R	PR	R	Cell proliferation; cellular aging; chaperone	Brain, Breast, colon, kidney, lung, ovarian [140]
139	GLU2B	Glucosidase 2 subunit beta	Score: 80 Mass: 59388 *pI*: 4.33 Coverage: 11 %	Endoplasmic reticulum	Up Regulated	OR	OR	-	Regulatory subunit of glucosidase II	Colon [138, 141]; sarcoma [142];
149	HSP74	Stress‐70 protein, mitochondrial	Score: 187 Mass: 94271 *pI*: 5.11 Coverage: 10 %	Cytoplasm	Down Regulated	-	OR	PR	Stress response; cell proliferation, differentiation	Breast [143]
170	HNRPC	Heterogeneous nuclear ribonucleoproteins	Score: 142 Mass: 33650 *pI*: 4.95 Coverage: 8 %	Nucleus	Up Regulated	-	R	OR	mRNA processing and translation	Breast [144]; colon [145]; lung [146]; pancreatic [147];
215	CALR	Calreticulin	Score: 246 Mass: 48112 *pI*: 4.29 Coverage: 17 %	Endoplasmic, sarcoplasmic reticulum	Up Regulated	-	R	OR	Calcium-binding chaperone	Bladder [69]; breast [148]; colon [149]; gastric [150]; glioblastoma [69]; liver [151]; ovarian [69]; pancreas [152]; prostate [153]
246	VIME	Vimentin	Score: 539 Mass: 53619 *pI*: 5.06 Coverage: 81 %	Cytoplasm	Up Regulated	P	OR	-	Filaments attached to nucleus and endoplasmic reticulum	Breast [154]; gastric [155]; lung [156]; pancreas [157]

(−) indicates that the spot was either not visible on the gel or that the treatment had no effect on the expression of the protein

*R* Restored, *PR* Partially Restored, *OR* Over Restored

## Discussion

In this study, the efficacy of administering CS, CB and OX in two aliquots with a time gap was investigated with the idea that results may provide information about the processes that take place during platinum-based chemotherapy practiced in the clinic. In particular, the results might provide valuable insight into the molecular aspect of administering the same drug in cycles. As noted earlier [11], among the three platinum drugs, OX was most active against the ovarian cancer A2780, A2780^cisR^ and A2780^ZD0473R^ cell lines but had the lowest activity against SKOV-3 cell line. The higher activity of OX as compared to CS against A2780, A2780^cisR^ and A2780^ZD0473R^ cell lines, could be due to differences in both the leaving groups and the carrier ligands in OX and CS (oxalate in OX as against chloride in CS and *trans-R,R*-diaminocyclohexane abbreviated as DACH in OX as against ammonia in CS). This difference allows several conformational differences to exist in the intrastrand 1,2-(GpG) adducts formed by CS and OX [16, 17]. Whereas the CS crosslink preferentially undergoes hydrogen bonding with the 5’ side of adduct (that causes a greater structural distortion to the base pair at the 5’ end), OX does so with the 3’ end of the intrastrand crosslink. Also, in the case of OX there is a strong hydrogen bond between the NH_2_ group of the DACH ligand and the O6 oxygen atom of the 3’ guanine nucleobase. It has been suggested that the conformational differences between OX − DNA and CS − DNA adducts may be responsible for differences in their protein recognition and cellular processing [16]. The low activity of OX against SKOV-3 cell line may lie in the p53-null status of the cells [18, 19].

As applied to the administration of CS, CB and OX in two aliquots with a time gap, experiments were done using both *fresh*/*fresh* and *fresh/aged* solutions. The purpose behind using both *fresh* and *aged* solutions was to determine the effect of speciation of activity of platinum drugs. Hydrolysis of platinum drugs produces highly reactive mono- and di-aquo species that can deprotonate and polymerise to produce species with multiple metal centres. The presence of the species is believed to alter the nature of interaction with DNA (as well as that with other cellular platinophiles). For example, multi-nuclear platinum species may carry a greater net positive charge making them more easily attracted to the negatively charged DNA. Furthermore, unlike CS that binds predominantly to one strand of DNA, multinuclear species may bind more significantly to both the strands of DNA [20, 21].

When CS, CB and OX were administered in two aliquots with a time gap than as a bolus, it was found that there was a greater cell kill with *fresh/aged* combination. The extent of cell kill was dependent on the duration of the time gap with 8 h time gap producing most pronounced cell kill in the parent A2780 cell line. In A2780^cisR^ and A2780^ZD0473R^ resistant cell lines, time gaps of 2, 4, and 8 h all caused pronounced cell death. In contrast, in SKOV-3 cell line, 24 h time gap was additive in action whereas 2 to 8 h time gaps were antagonistic. Additiveness to antagonism observed with *fresh/fresh* combinations and synergism seen with *fresh/aged* combinations, suggest that the increased cell kill associated with the latter may be due to the formation of more cytotoxic species resulting from hydrolysis, deprotonation and polymerisation reactions and the rates of these processes are considered to be greater at the ambient temperature than under the frozen condition. In line with the idea, it was found that when solutions of CS were left standing at room temperature, the cell killing effect increased with time (data not shown) as was observed by others (Yachnin [21]). To gain a better understanding of the speciation of platinum drugs in solution in terms of hydrolysis and formation of more cytotoxic species, limited mass spectral measurements with solutions of OX were carried out. The results are discussed latter in the paper.

Also whether the enhanced cell kill due to the administration of platinum drugs in two aliquots with a time gap was associated with a corresponding increase in cellular accumulation of platinum and consequently a greater level of platinum − DNA binding or it was due to speciation of platinum drugs in cell culture medium at the ambient temperature, the cellular platinum accumulation and platinum − DNA binding levels associated with the administration of CS in two aliquots with a time gap (2 and 4 h) were determined, as applied to both *fresh/fresh* and *fresh/aged* combinations. It may be noted that although platinum − DNA binding is a necessary step in the programmed cell death due to platinum drugs, it is not sufficient as the programmed cell death is actually carried out by downstream processes in the cell cycle in which many proteins are involved. In any case, the drugs must enter the cells before they can bind with DNA or be deactivated due to binding with cellular platinophiles [3, 22].

Figures 4 and 5 gave the cellular accumulation and platinum − DNA binding levels resulting from administration of CS in two aliquots with time gaps of 2 and 4 h in A2780 and A2780^cisR^ cells, as applied to both *fresh/fresh* and *fresh/aged* combinations. It was found that *fresh*/*aged* combination produced greater platinum accumulation than the *fresh/fresh* combination suggesting that there were either greater influx or reduced efflux or both. Since the species formed are likely to be positively charged, they are more likely to be transported by organic cationic transporters rather than by passive diffusion and copper transporter 1 that is also known to transport CS into the cell (CTR1) [23]. As applied to platinum − DNA binding level, it was found that in the parent A2780 cell line administration in two aliquots with a time gap resulted in a lower platinum − DNA binding level with *fresh/fresh* combination than the bolus but a greater level from *fresh*/*aged* combination. In the CS-resistant A2780^cisR^ cell line, both *fresh/fresh* and *fresh/aged* combinations resulted in significantly greater platinum − DNA binding levels than the bolus. The results indicate that (regardless of the ageing status of the solution) the administration of platinum drugs in two aliquots with a time gap may be better able to overcome mechanism of platinum resistance in the CS-resistant cell line, giving support to the merit of cycle regimens used in the clinic.

Cellular glutathione levels were also determined as a measure of changes in redox status of the cell. Figure 6a and b respectively gave the levels of total glutathione (GSH and GSSG) and oxidized glutathione (GSSG) in A2780 and A2780^cisR^ cell lines following their treatments with solutions of CS and CB administered as a bolus and in two aliquots with a 4 h time gap. Although the level of total glutathione in both A2780 and A2780^cisR^ cell lines after drug treatments was lower than the levels before treatment (irrespective of whether the drug was administered as a bolus or in two aliquots), the decrease was greater for the bolus than the administrations in two aliquots. CB was found to be more effective than CS in lowering the level of GSH in the CS-resistant A2780^cisR^ cell line although a kinetic study suggested that rates of reaction CS and OX with GSH were 5-fold greater than that of CB [24].

A smaller decrease in GSH level observed in both A2780 and A2780^cisR^ cells after treatment with CS and CB given as a bolus than in two aliquots (with a time gap), rebuts the hypothesis that the first aliquot would decrease glutathione level enabling more of the second aliquot of platinum to bind to DNA. This means that the greater cell kill resulting from administration of platinum drugs in two aliquots with a time gap can be due to speciation of platinum drugs rather than changes in cellular glutathione level. Thus, other changes such as differential expression of proteins may be responsible for enhanced cell kill. The level of oxidised glutathione (GSSG) is also found to decrease following treatment of cells with CS and CB suggesting enhanced efflux of conjugated products through MRPs.

Although in this study the mass spectral analysis of platinum speciation has been attempted primarily to find out about speciation of platinums on *ageing* i.e. when left standing at ambient temperature, it is important to note that platinum speciation especially in solution in the biological matrix gained increasing focus when it became clear that (1) the active drugs were the hydrolysed products rather than the intact molecules and that (2) the drugs are inactivated due to conjugation with proteins and peptides (such as glutathione) [25]. When solutions of OX made in buffered cell culture media were left standing at room temperature, a number of peaks were observed indicating that OX in solution underwent hydrolysis followed by further speciation. It should however be noted that many possible matches in terms of structure could be found for a given peak in the mass spectrum (having defined mass to charge ratio, mass error of less than 2 ppm and isotopic splitting pattern). For example the peak with m/z = 420 had four possible matches whereas that with m/z = 940 had 377 matches. Notwithstanding the multitudes of structural possibilities for the observed peaks, it can be seen that OX in solution had underwent hydrolysis and that the products of hydrolysis conjugated with components present in the cell culture media. A detailed description of the peaks in terms of suggested structures is given as follows. It should be noted when the drug enters the cell, other compounds are likely to be formed due to binding with the cellular constituents. For example, formation of inactive complexes such as Pt(DACH)(GSH), Pt(DACH)(Cys) and Pt(DACH)(Met) due to binding of the Pt(DACH) unit with cellular constituents GSH, Cys and methaionine (Met) have been reported [26]. The adducts may be inactive if they are inert towards binding with DNA. However, formation of such adducts within the cell may lead to oxidative stress and the resulting side effects due to depletion of cellular thiols [27]. A more definitive statement about the cytotoxicity of adducts can be made when they are isolated using a suitable method of separation e.g. reverse phase HPLC. It is possible that dimeric species Pt_2_(C_6_H_14_N_2_)_2_(C_2_O_4_)_2_ can be a potent DNA binder with enhanced cytotoxicity. It is appropriate to note that many more dimeric and trimeric species were formed in the *aged* solution of OX in mQ water i.e. in the absence of culture media than in its presence. One important difference between speciation of OX in the buffered cell culture media and in solution in mQ water was that a greater number of hydrolysed products e.g. hydroxy, aqua and oxalate bridged dimeric, trimeric, tetrameric and even pentameric species were formed in the *aged* solution of OX in milli-Q water. The paucity of such multimeric species in the cell culture media, may indicate that complexation with constituents of the media had served to hinder the polymerisation reactions. It is also possible that the background noise in the spectra might have served to mask their presence. Finally, the presence of dimeric species was also reported in the *aged* solution of cisplatin [28].

### Proteomics

Proteomics involving 2-D gel electrophoresis and mass spectrometry were employed to identify key proteins associated with drug resistance in A2780^cisR^ ovarian cancer cell line. It was based on the idea that proteins associated with drug resistance would undergo marked changes in expression in the resistant A2780^cisR^ cell line as compared to that in the parent A2780 cell line. It was also thought that the proteins in question might be restored back to normalcy after treatment with drugs in two aliquots that caused enhanced cell kill (although the involvement of multiple pathways both in apoptosis and cell survival may mean not all of the proteins need to be concurrently targeted to bring about the cell death). However, the difficulty in the extraction of hydrophobic proteins including transmembrane proteins means that the identification of proteins such as CTR1 and other platinum influx transporters may remain elusive. Furthermore, proteomic results provide only a static picture at a selected time point (or points) whereas the cells being dynamic in nature would undergo continual changes. Other drawbacks of proteomics include difficulty in extraction of proteins that are low in abundance and inability to provide a complete picture at the levels of organs and organisms [29]. Notwithstanding these limitations, in this study 390 proteins were identified of which 72 underwent significant changes in expression in the resistant A2780^cisR^ cells compared to the levels found in the parent A2780 cells. Administration of CS in two aliquots with a time gap restored the expressions of at least 22 proteins to levels comparable to those found in the parent cell line, of which 12 were down-regulated and 10 up-regulated prior to drug treatment. A summary of the proteins, their possible functions, and associations with neoplasia were given in Table 4.

One of the proteins undergoing differential expression is mitochondrial cytochrome c oxidase subunit 5A (COX5A) which is one of the 13 subunits that make up cytochrome c oxidase (COX), the terminal enzyme of the mitochondrial electron transport chain. It was down-regulated in untreated A2780^cisR^ cell line as compared to the parent A2780 cell line, over-restored due to treatment with *aged* solution of CS administered in two aliquots with 2 h time gap but only partially-restored when the same treatment was given with *fresh* solution. Although the three largest subunits COX1, COX2 and COX3 form the catalytic core of cytochrome oxidase, COX5A coupled to COX4 is essential for the assembly of the entire unit [30] so that a decrease in the expression of COX5A may be an indicator of under performance of the entire enzyme. COX5A was found to be down-regulated in other cancers such as nasopharyngeal and gastric carcinomas [31, 32]. Thus, the up-regulation of COX5A in A2780^cisR^ cells following treatment with solutions of CS administered in two aliquots with a time gap can be a factor responsible for the pronounced cell kill. Cofilin-2 (COF-2) was also down-regulated in A2780^cisR^ cell line as compared to the parent A2780 cell line. Administration of *fresh* solution of CS in two aliquots with a 2 h time gap caused its full restoration whereas that with the *aged* solution led to over-restoration. The results indicate that COF-2, which is the major component of intranuclear and cytoplasmic actin rods that plays a critical role in the regulation of actin filament dynamics in eukaryotes [33], may be associated with platinum resistance in ovarian cancer and that the employed treatments have been able to overcome the mechanism of resistance applying to the protein. Another protein found to be down-regulated in A2780^cisR^ as compared to that in A2780 cell line was 14-3-3γ. Present abundantly in the cytoplasm, 14-3-3 proteins participate in a wide variety of activities including DNA repair, apoptosis, the onset of cell differentiation and senescence, and the coordination of cell adhesion, motility, intracellular signalling and cell cycle control [34]. Administration of CS in two aliquots with 2 h time gap caused its partial restoration when *fresh* solutions were used but led to over-restoration with *aged* solutions. The results indicate that 14-3-3γ may be a key protein associated with platinum resistance in ovarian cancer. Another protein of interest is MARE1 that belongs to the microtubule-associated protein RP/EB family. It is a prototypic member of microtubule plus-end tracking proteins (+TIPs) that control microtubule dynamics and are associated with different cellular structures. MARE1 was down-regulated in A2780^cisR^ ovarian cancer cell line as compared to A2780 cell line. The administration of CS in two aliquots with 2 h time gap led to its full restoration. Although down-regulation of MARE1 inhibits the formation of stable microtubule, the role of MARE1 in inducing chromosomal instability remains unclear [35]. The next identified protein was Annexin A1 that belongs to the annexin family of proteins that have been implicated in many molecular and cellular processes, including modulation of phospholipase A2 and kinase activities in signal transduction, maintenance of cytoskeleton and extracellular matrix integrity, tissue growth and differentiation, inflammation, and blood coagulation [36–38]. ANXA1 was found to be down-regulated in the CS-resistant A2780^cisR^ cell line as compared to the parent A2780 cell line. The administration of CS in two aliquots with 2 h time gap fully restored ANXA1. The results indicate that ANXA1 may be playing an important role in drug resistance in ovarian cancer and that the administered combinations have been able to overcome associated mechanism of resistance. Another protein found to be down-regulated in the CS-resistant A2780^cisR^ cell line, was nucleophosmin (NPM), also known as nucleolar phosphoprotein B23. The administration of CS in two aliquots caused its full restoration when *fresh* solutions were used but caused its over-restoration with the *aged* solution. The over-expression of the protein in A2780^cisR^ cells due to treatment with the *aged* solution can be a reason why a greater cell kill was produced from the *aged* solution than *fresh* counterpart. A number of studies suggest that NPM is involved in cancer pathogenesis. In mice, inactivation of NPM in the germ line leads to a host of developmental defects that cause embryonic lethality at mid-gestation. Haploid-insufficiency of NPM leads to unrestricted centrosome duplication and genomic instability with mice developing myelodysplasia with an acceleration of oncogenesis [39]. Moreover, disruption of the NPM gene by translocation is frequently found in human hematopoietic malignancies [40]. The fact that NPM contributes to oncogenesis by activating the oncogenic potential of the fused protein partner, suggests that the down-regulation of NPM may also indicate the under-regulation of the tumour suppressors p53, Rb and ARF [41, 42]. Alpha-enolase (ENOA) was also down-regulated in A2780^cisR^ cell line as compared to the parent A2780 cell line. The protein acts as a transcriptional repressor and possibly functions as a tumour suppressor. Partial restoration occurred when A2780^cisR^ cells were treated with *fresh* solutions of CS administered in two aliquots with 2 h time gap whereas the same with *aged* solutions caused its full restoration. The results indicate that the treatments in two aliquots using *aged* solutions of CS were able to overcome mechanism of resistance associated with ENOA. Another protein found to be down-regulated in CS-resistant A2780^cisR^ cell line as compared to its CS sensitive counterpart was PDIA3. Partial and over-restoration of PDIA3 occurred when cells were treated respectively with *fresh* and *aged* solutions of CS administered in two aliquots. Although the expression of PDIA was found to be up-regulated in some cancers such as breast, it was found to be down-regulated in other cancers such as gastric and prostate cancers [43–46]. Besides its role as a chaperone, it was suggested that PDIA3 might be functioning as a pro-apoptotic protein in prostate cancer; a decrease of caspase activity was related to due to down-regulation of PDIA3 in prostate cancer cell lines [46]. Down-regulation of PDIA3 might be playing a role in the late onset of prostate cancer progression. Down-regulation of PDIA3 also correlated with increased tumour invasion and advanced stage of gastric cancer. Hence PDIA3 has been proposed to be a negative prognostic marker [45]. In addition to its role in the ER stress pathway, PDIA3 has also gained attention due to its association with the major histocompatibility complex (MHC) class I pathway. In PDIA3 deficient mice, MHC I is impaired and negatively influences presentation of antigenic peptides helping tumours to escape from immune surveillance by cytotoxic T cells [47]. Although PDIA3 was found to be over-expressed in YDOV-139 ovarian cancer cell line [48], in this study, CS-resistant ovarian cancer cell line showed decreased levels of the protein compared to the parent cell line suggesting its involvement in resistance to cisplatin. As PDIA3 is reported to be up-regulated by hypoxia [49], low levels of PDIA3 found in A2780^cisR^ cells indicate that the cells may not be under hypoxic stress.

Calumenin (CALU) was also up-regulated in the resistant A2780^cisR^ as compared to that in A2780 cell line. It is a ubiquitous calcium-binding protein localized in the endoplasmic reticulum and involved in such functions as protein folding and sorting [50]. Although the exact role of CALU is yet to be elucidated, the functions of the calcium-binding family are well understood. They have been associated with resistance to chemotherapeutic drugs [51, 52]. In this study, administration of *fresh* solution of CS in two aliquots with a 2 h time gap caused its over-restoration whereas that with *aged* solution of CS led to its partial-restoration. The results indicate that CALU may be associated with drug resistance and that the employed drug combinations have been able to overcome the associated mechanism. Another protein that was up-regulated in the A2780^cisR^ as compared to the parent A2780 cell line was the 40S ribosomal protein SA (RSSA). The administration of CS in two aliquots using *fresh* solution fully restored its expression. The elevated expression of RSSA in the resistant A2780^cisR^ cell line and its restoration back to normalcy due to synergistic treatments give support to the idea that the receptor is associated with metastasis and drug resistance. Laminin has been implicated in a wide variety of biological processes including cell adhesion, differentiation, migration, signalling, neurite outgrowth and metastasis. The protein also serves as a major adhesion substrate for invasive cancer cells. Indeed, there is a direct correlation between the ability of malignant cells to attach to laminin and their metastatic potential [53]. Thus, over-expression of RSSA was observed in many cancers indicating its potential role in tumour progression. Highly metastatic cancer cells are found to express at their surface significantly more laminin receptors than do their much less metastatic or benign counterparts [54]. Breast, cervical, colorectal and gastric carcinomas are found to express high levels of RSSA [54, 55]. Over-expression of this receptor is not restricted to epithelial tumours. For example, melanomas and lymphomas also display increased expression of the receptor [56, 57]. Next protein that was up-regulated in A2780^cisR^ as compared to A2780 cell line was ACTB that is a member of the actin family. Actins are essential for a large range of cell functions including cell division, migration, junction formation, chromatin remodelling, transcriptional regulation, vesicle trafficking, and cell shape regulation [58]. Administration of CS in two aliquots using *fresh* solution caused its full restoration in A2780^cisR^ cell line but partial restoration when *aged* solutions were used. The results indicate the employed treatments were able to overcome the associated mechanism of resistance. Three HNRNP proteins identified in this study were HNRPC, HNRPF and HNRPK. HNRPF was down-regulated whereas HNRPC and HNRPK were up-regulated in the A2780^cisR^ cell line compared to the parent A2780 cell line. Although the proteins were differentially expressed in the resistant cell line, there does not appear to be a direct relationship between HNRNPs and cellular resistance to platinum drugs. ATPA and ATPB were up-regulated in the CS-resistant A2780^cisR^ cell line as compared to the parent A2780 cell line. Administration of both *fresh* and *aged* solutions of CS in two aliquots with 2 h time gap caused over-restoration of ATPB. ATP synthase has been implicated in angiogenesis, cellular immunity, cholesterol uptake and cellular pH regulation [59]. A novel approach by Juan et al. targeted the deregulation and over-expression of ATP synthase in breast cancer using an ATP synthase inhibitor [60]. It may be noted that the spot corresponding to ATPA was positioned in a cluster of spots that were closely placed and were of poor quality after treatment so that some uncertainty remained about ATPA. Further experiments would be needed to ascertain changes in expression of ATPA. However, ATP synthase appears to be associated with drug resistance and that the employed treatments were able to completely inhibit the expression of ATPB. The protein coded as T-complex protein 1 subunit theta (TCPQ/CCTθ) was down-regulated in A2780^cisR^ as compared to the level found in parent A2780 cell line. Although evidence is emerging about the diverse roles played by the complex, relatively little is known about the functional divergence of the individual subunits, and how this may relate their role in tumour development and progression. Treatment with solutions of CS given in two aliquots with 2 h time gap caused its over-restoration with *fresh* solution and partial restoration with *aged* solution. Another protein glucosidase 2 subunit beta coded as GLU2B was up-regulated in the A2780^cisR^ cell line as compared to the A2780 cell line. Though the exact function of GLU2B in cell differentiation is yet to be determined, it may be influencing glycosylation process of newly synthesized proteins and may act as a regulator of distinct developmental processes [61]. It was reported by Otto Warburg about 70 years ago that tumour cells exhibited an altered metabolism, characterized by increased glucose uptake and elevated glycolysis [62]. Indeed, an increase in the rate of glycolysis is one of the metabolic alterations found in most cancer cells [63, 64]. GLU2B was over-restored in A2780^cisR^ cell line due to the administration of *fresh* solution of CS given in two aliquots with a time gap but the same with *aged* solutions had no effect on the expression of GLU2B. The results suggest that GLU2 may be associated with drug resistance in ovarian cancer although the enhanced cell kill associated with *aged* solutions cannot be related to the protein. Calreticulin (CALR) that is a multifunctional protein that acts as a major calcium-binding protein in the lumen of the endoplasmic reticulum [65], was up-regulated in the CS-resistant A2780^cisR^ cell line as compared to the parent A2780 cell. It is involved in a wide variety of cellular processes including modulation of calcium signals, storage and buffering of calcium, regulation of steroid-dependent gene expression via direct interaction with steroid receptors, cell adhesion via direct binding to integrin α, a chaperone in protein folding, autoimmune response and long-term neuromodulations [66]. Down-regulation of CALR by antisense was found to increase sensitivity of neuroblastoma × glioma NG-108–15 cells to cytotoxic calcium overload [67]. In contrast, up-regulation of CALR has been shown to protect HeLa cells from apoptosis [66]. Nakajo et al. reported that the expression of CALR markedly decreased before the apoptosis event in human leukemia HL-60 cells [68]. Furthermore, increased expression of CALR was a poor prognostic factor in diverse tumours including neuroblastoma, bladder cancer, and non-Hodgkin’s lymphoma [69]. Interestingly, CALR is thought to function as a pro-phagocytic signal highly expressed on the surface of several human cancers, but minimally expressed on normal cell counterparts line [69]. The protein was fully-restored due to treatment with *fresh* solutions of CS given in two aliquots with a time gap whereas treatment with the *aged* solutions caused its over-restoration. The results can be seen to indicate that CALR may be associated with platinum resistance in ovarian cancer. Next protein that was found to be up-regulated in CS-resistant A2780^cisR^ cell line as compared to the parent A2780 cell line was vimentin (VIME). VIME is ubiquitously expressed in normal mesenchymal cells and known to maintain cellular integrity and provide resistance against stress. Increased expression of VIME has been reported in many epithelial cancers including melanoma, prostate, gastric, oesophageal, hepatocellular, pancreatic and breast carcinomas [70–73]. The over-expression of VIME in cancer is extensively reported to correlate with increased tumour growth, invasion, metastasis and poor prognosis [74]. In contrast, down-regulation of VIME inhibits carcinoma cell migration and adhesion [75]. VIME has gained much importance as a marker of epithelial-mesenchymal transition (EMT); a cellular reprogramming process in which the epithelial cells acquire a mesenchymal phenotype that renders the cells to significantly alter their shape and show increased motility [76]. It was over-restored due to treatment with *fresh* solutions of CS administered in two aliquots with a time gap whereas the same with the *aged* solutions had no significant effect on its expression. The results suggest that VIME may be associated with platinum resistance and that the increase in its expression may be associated with poor prognosis in platinum refractory ovarian cancer. A number of heat shock proteins (HSP) were also found to be differentially expressed in A2780^cisR^ cell line as compared to A2780 cell line. The first was chaperonin 60 kDa (CH60) also known as HSP60. It was up-regulated in A2780^cisR^ cell line as compared to A2780 cell line. The protein was restored back when the cells were treated with solutions of CS administered in two aliquots – over-restored in the case of *fresh* solution and partially restored in the case of *aged* solution. The results indicate that the over-expression of HSP60 may provide the cells with survival advantage by making them more tolerant against drugs and that the employed drug treatments were able to overcome the associated mechanism of resistance. Three HSPs belonging to the HSP-70 family (HSP-70s) namely HSP7C, HSP74 and GRP75 were also identified in the study. HSP74 was down regulated in A2780^cisR^ cell line as compared to the parent A2780 cell line. It was over-restored due to treatment with *fresh* solution of CS administered in two aliquots and partially restored when the same was given using *aged* solution. HSP7C did not show any change in expression in A2780^cisR^ cell line as compared to A2780 cell line. The results of the study show that whereas HSP74 may be associated with CS resistance in the tested ovarian cancer cell lines, HSP7C may not be so. GRP75 was also slightly down-regulated in A2780^cisR^ as compared to A2780 cell line. It was partially and fully-restored when the cells were treated with *fresh* and *aged* solutions of CS administered in two aliquots respectively. The expression pattern of GRP75 suggests that the protein may not be a major player in CS resistance.

In summary belongingness of the 22 identified proteins to various functional groupings such as invasion and metastasis, cell cycle regulation and proliferation, metabolic and biosynthesis processes, stress-related proteins and chaperones, mRNA processing, cellular organization/cytoskeleton, cellular communication and signal transduction highlights that platinum resistance is multifactorial in nature in which many proteins with diverse functions may be playing key roles; inevitably the loss of control of functions can endow tumour cells with the ability to escape programmed cell death and proliferate without control. The results also indicate that multiple strategies can be gainfully employed to overcome the resistance.

### Putting into context

With the idea that the effect of administration of platinum drugs in two aliquots with a time gap may induce changes in multiple parameters, in addition to changes in the combined drug action, cellular accumulation of platinum, level of platinum − DNA binding, cellular glutathione level and changes in protein expression were also determined. Whereas the combined drug action was quantified for all aliquoted administrations, the cellular accumulation of platinum, level of platinum − DNA, cellular glutathione levels and proteomic studies were carried out for a subset of the experiments. It was thought that a careful consideration of the results for the subset might lead to more meaningful conclusions. Table 5 provides a summary of all the results for the subset.

**Table 5:** Summary of results following treatment of A2780^cisR^ cell line with CS administered in two aliquots with 2 h time gap

	Cytotoxic effect		Cellular accumulation		DNAbinding		Proteomics^a^			
	Fresh	Aged	Fresh	Aged	Fresh	Aged	Fresh		Aged	
*CS + CS*										
2 h	Antagonistic	Synergistic	Unchanged	Increased	Increased	Greater increase	**COF2** **MARE1** **NPM** **ANXA1** **RSSA** **ACTB** **CALU** **HNRPF** **ATPA** **HNRPK** **CH6**0 **TCPQ** **GLU2B** **HSP74** **HNRPC** **CALR** **VIME**	(D,R), (D,R), (D,R), (D,R), (U,R), (U,R), (U,OR), (D,R), (U,OR), (U,OR), (U,OR), (D,OR), (U,OR), (D,OR), (U,R), (U,R), (U,OR).	**COX5A** **COF2** **1433γ** **MARE1** **NPM** **ANXA1** **HNRPF** **ENOA** **ATPA** **PDIA3** **TCPQ** **GRP75** **HNRPC** **CALR**	(D,OR), (D,OR), (D,OR), (D,OR), (D,OR), (D,OR), (D,OR), (D,R), (U,OR), (D,R), (D,R), (D,R), (U,R), (U,OR).

^a^The proteins listed have been fully/over restored in A2780^cisR^ due to the selected combinations as compared to the parent A2780 cell line. Keys: U = up-regulated, D = down-regulated, OR = over-restored, R = fully-restored

Although treatment with *fresh* solution was found to be less synergistic to antagonistic (whereas that with *aged* solution was more synergistic), both the cellular accumulation of platinum and the level of platinum − DNA binding were elevated due to treatment of A2780^cisR^ cells with the *fresh* solution of CS in two aliquots. The failure of the increased cellular accumulation of platinum and more importantly that of the increased level of platinum − DNA binding to translate into enhanced cell death highlights the fact that although platinum − DNA binding can be a necessary step towards programmed cell death, it is not sufficient as apoptosis is brought about by downstream processes in the cell cycle in which many proteins may be playing key roles. The presence of crosstalk between pro-apoptotic and anti-apoptotic pathways can also be seen to complicate the situation.

**Fig. 10:**
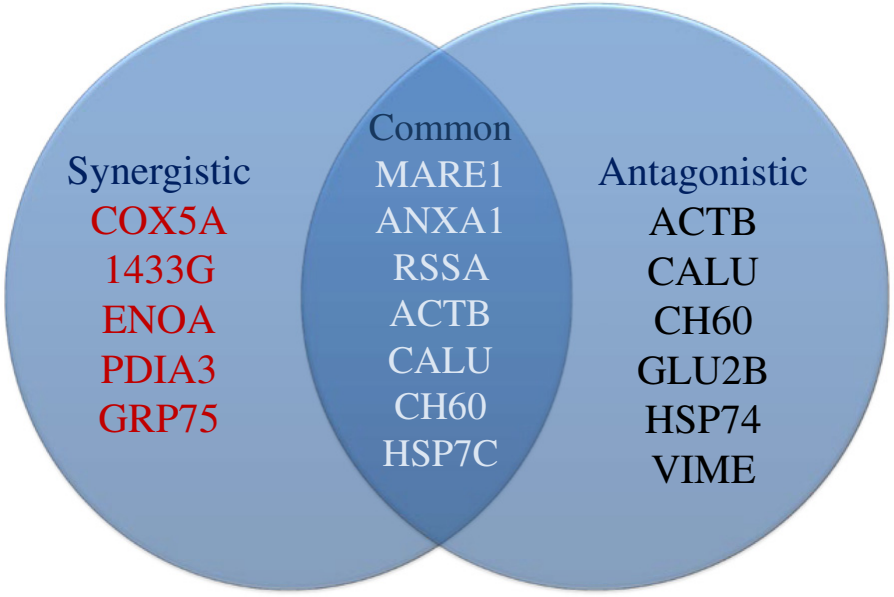
Venn diagram listing the proteins expressed in A2780^cisR^ cells that were restored or over restored compared to the levels found in A2780 cells after treatment of cells with *aged* (synergistic) and *fresh* (antagonistic) solutions of CS in two aliquots with 2 h time gap

For the antagonistic administration of CS in two aliquots using *fresh* solution, the following proteins: ACTB, CALU, CH60, GLU2B, HSP74 and VIME in treated A2780^cisR^ cells were fully or over restored to the levels found in the parent A2780 cell line. In contrast, for the administration of CS in two aliquots using *aged* solution which was synergistic in action, the following proteins: COX5A, 1433γ, ENOA, ATPA, PDIA3 and GRP75 were fully or over restored in treated A2780^cisR^ cells as compared to the levels found in untreated A2780 cells. This is illustrated in the Venn diagram below (Fig. 9) where the proteins listed in red namely COX5A, 1433γ, ENOA, PDIA3 and GRP75 are considered to be characteristic of incubation of cells with *aged* solution CS in two aliquots with 2 h time gap (Fig. 10).

COX5A, 1433G, ENOA, PDIA3 and GRP75 that are characteristic of synergistic administration of *aged* solution CS in two aliquots with 2 h time gap were initially down-regulated in the resistant cell line but up-regulated following the drug treatment indicating that these proteins may be playing a pro-apoptotic role so that their down-regulation served to dampen cell death. In contrast, the proteins: MARE1, ANXA1, RSSA, ACTB, CALU, CH60 and HSP7C that were restored due to treatment with both synergistic and antagonistic combinations, may not be so critically associated with synergistic drug action in spite of them being a hallmark of cancer cell biology and platinum drug resistance. The results of the present study can be seen to confirm that synergistic administration of drugs may provide a means of overcoming drug resistance due to the reestablishment of cellular control functions.

## Conclusion

The results of the present study show that when platinum drugs are administered in two aliquots with a time gap, a greater cell kill is produced from treatment with *aged* solutions than that with *fresh* ones. A smaller decrease in cellular GSH level in both A2780 and A2780^cisR^ cells after treatment with CS and CB given in two aliquots than as a bolus, indicates that the increased activity resulting from administration in two aliquots cannot be due to changes in GSH. The increased activity on *ageing* is believed to be related to speciation of the drug in solution. Proteomic studies have identified 72 proteins that were differentially expressed in A2780 and A2780^cisR^ cell lines, 22 of them were restored back to the levels found in the parent cell line as a result of synergistic treatments, indicating their relevance in synergistic drug action. Among them COX5A, 1433G, ENOA, PDIA3 and GRP75 that were down-regulated in the resistant A2780^cisR^ cell line as compared to that in parent A2780 cell line but up-regulated after synergistic treatments, are considered to play a more critical role in bringing about apoptotic cell death. In contrast, MARE1, ANXA1, RSSA, ACTB, CALU, CH60 and HSP7C which were restored due to treatment with both synergistic and antagonistic combinations, may not be so critically involved in apoptosis or escape from it, in spite of them being a hallmark of cancer cell biology and platinum drug resistance. Finally, it should be stated that a major limitation of the study is that it gives a static picture. However, cells are dynamic in which changes (especially for proteins) are constant.

## Abbreviations

ACN: Acetonitrile
ACTB: Actin, cytoplasmic 1
ANXA1: Annexin A1
ATPA: ATP synthase subunit alpha, mitochondrial
CALR: Calreticulin
CALU: Calumenin
CB: Carboplatin
CBDCA: Cyclobutanedicarboxylate
CH60: 60 kDa heat shock protein, mitochondrial
CI: Combination index
CID: Collision induced dissociation
COF2: Cofilin-2
COX5A: Cytochrome c oxidase subunit 5A, mitochondrial
CS: Cisplatin
CTR1: Copper transporter 1
Cu: Copper
DACH: Diaminocyclohexane
ENOA: Alpha-enolase
FCS: Foetal calf serum
GLU2B: Glucosidase 2 subunit beta
GRP75: Stress 70 protein, mitochondrial
GSH: Glutathione
GSSG: Oxidised glutathione
HCL: Hydrochloric Acid
His: Histidine
HNRP: Heterogeneous nuclear ribonucleoproteins
HSP74: Stress-70 protein, mitochondrial
IEF: Isoelectric focusing
IkB: Inhibitor of Kappa B
MALDI-MS: Matrix assisted laser desorption ionisation mass spectrometry
MARE1: Associated protein
MTT: 3-(4,5-Dimethylthiazol-2-yl)-2,5-diphenyltetrazolium bromide
Nd:YAG: Neodymium-doped yttrium aluminum garnet
NF-kB: Nuclear factor Kappa B
NPM: Nucleophosmin
OX: Oxaliplatin
MAPRE1: Microtubule-associated protein RP/EB family member 1
Met: Methionine
MRP: Multidrug resistance
PDIA3: Protein disulfide-isomerase A3
PBS: Phosphate buffered saline
Pt: Platinum
Pyr: Pyridoxine (vitamin B6)
ROS: Reactive oxygen species
RSSA: 40S ribosomal protein SA
SDS: Sodium dodecyl sulfate
TCPQ: T complex protein 1 subunit theta
TFA: Trifluoroacetic acid
VIME: Vimentin
1433G: 14-3-3 protein gamma
